# Protective Effect of Conditioned Medium of Immortalized Human Stem Cells from Exfoliated Deciduous Teeth Against Hair Graying Caused by X-Ray Irradiation via Its Antioxidative Activity

**DOI:** 10.3390/antiox14010109

**Published:** 2025-01-18

**Authors:** Yasuhiro Katahira, Eri Horio, Natsuki Yamaguchi, Jukito Sonoda, Miu Yamagishi, Satomi Miyakawa, Fumihiro Murakami, Hideaki Hasegawa, Izuru Mizoguchi, Takayuki Yoshimoto

**Affiliations:** Department of Immunoregulation, Institute of Medical Science, Tokyo Medical University, 6-1-1 Shinjuku, Shinjuku-ku, Tokyo 160-8402, Japan; yasuhiro@tokyo-med.ac.jp (Y.K.);

**Keywords:** cell-free therapy, conditioned medium, hair graying, antioxidative activity, stem cells from human exfoliated deciduous teeth

## Abstract

Hair graying is one of the common visible signs of human aging, resulting from decreased or abolished melanogenesis due to the depletion of melanocyte stem cells through excess accumulation of oxidative stress. Cell-free therapy using a conditioned medium (CM) of mesenchymal stem cells has been highlighted in the field of regenerative medicine owing to its potent therapeutic effects with lower regulatory hurdles and safety risk. Recently, we demonstrated that a CM of an immortalized stem cell line from human exfoliated deciduous teeth (SHED) has protective effects against a mouse model of ulcer formation via antioxidative and angiogenic activities mediated by HGF and VEGF. However, to date, no effective treatments for hair graying have been developed, and the effect of SHED-CM on hair graying remains unknown. In this study, we have investigated the effect of SHED-CM on a hair graying mouse model caused by X-ray irradiation. Repetitive subcutaneous administrations of SHED-CM greatly suppressed the development of hair graying, when compared to control medium, resulting in reduced cutaneous expression of 8-hydroxy-2′-deoxyguanosine, the major product of DNA damage induced by reactive oxygen species. Consistent with these in vivo results, SHED-CM significantly inhibited the cell death caused by X-ray irradiation in melanoma cell line B16F10 cells. Immunodepletion of HGF or VEGF in the SHED-CM revealed that this inhibition was due to suppression of the generation of reactive oxygen species, which was mainly mediated by HGF and probably VEGF. These results suggest that SHED-CM has protective effects against hair graying via its antioxidative activity.

## 1. Introduction

Hair graying is a common visible sign of human aging that has marked aesthetic and psychological impacts on appearance, thus affecting the confidence, self-esteem, and quality of life of individuals. Hair color is generated by melanin produced in melanocytes, and its graying is caused by decreased melanogenesis due to exhaustion of the pigmentary function of melanocytes in the hair bulbs [1,2,3]. This is attributed to the depletion of bulge melanocyte stem cells (McSCs)—which have self-renewal and multipotency abilities—through various factors, including genetics, hormonal changes, stress, inflammation, nutritional factors, etc. [4,5,6]. Melanogenesis is the formation of melanin in melanocytes through various reactions [7] and is accompanied by the generation of high levels of reactive oxygen species (ROS), which need to be scavenged by antioxidant mechanisms including catalase, superoxide dismutase, nuclear factor erythroid 2-related factor 2, and eumelanin synthesis. However, aging impairs antioxidant mechanisms, and the consequent excess accumulation of ROS stimulates McSCs and induces their vigorous proliferation, eventually depleting them [8]. To date, no effective treatments for hair graying have been developed [9]. Generally, people with hair graying will utilize colorants or dyes if they wish to recolor their hair. However, commercially available permanent colorants and dyes are often toxic and damage the hair and skin; however, people must continuously repeat the hair coloring process with the increasing risk of developing diseases such as contact dermatitis [10]. Therefore, safer and effective treatments targeting the biological processes of melanogenesis for the re-pigmentation of hair are highly desired.

With recent advances in regenerative medicine, accumulating evidence has revealed that cell transfer therapy using mesenchymal stem cells (MSCs)—adult stem cells with self-renewal and multipotent differentiation capacities [11,12,13]—has potent therapeutic effects against various diseases, including autoimmune diseases, inflammatory diseases, and bone and neurological disorders, through their tissue repair and regeneration potential and immunomodulatory ability [14]. Although MSCs present multipotency, various studies have revealed that the therapeutic effects of MSCs are highly attributed to the paracrine effects through secretion of various bioactive factors, including cytokines, growth factors, and exosomes [15,16,17]. Therefore, in place of MSC transfer therapy, cell-free therapy using its conditioned medium (MSC-CM) has been highlighted as having several advantages. Cell-free therapy raises much less concern regarding ethical and regulatory issues, as well as safety risks such as immunogenicity, embolism, thrombosis, or tumor progression [18,19,20]. Moreover, therapies using MSCs have been demonstrated to be therapeutically effective against hair loss and androgenetic alopecia in patients and animal models [21,22,23]. Nevertheless, to the best of our knowledge, there has only been one report showing the effectiveness of cell transfer therapy of MSCs on the reversal of hair graying in elderly patients [24], and there have been no reports on the effectiveness of MSC-CM on hair graying to date. Therefore, further investigation into the effects of cell transfer therapy of MSCs and cell-free therapy using MSC-CM against hair graying is necessary.

We have recently generated an immortalized stem cell line from human exfoliated deciduous teeth (SHED) [25]. We demonstrated that the immortalized SHED constantly secretes abundant cytokines, and that the SHED-CM had potent therapeutic effects in a mouse model of the formation of pressure ulcer induced by cutaneous ischemia–reperfusion injury [25]. This therapeutic effect highly depends on antioxidative and angiogenic activities, mainly via vascular endothelial growth factor (VEGF) and hepatocyte growth factor (HGF). As hair graying is highly attributable to oxidative stress and SHED-CM has potent antioxidative activity, we have herein investigated the therapeutic effects of SHED-CM on a mouse model of hair graying caused by X-ray irradiation [26,27]. The aim of this study was to determine whether SHED-CM has a protective effect against hair graying. This mouse model is suitable for the study of DNA damage, oxidative stress, and cell death induced by ionizing radiation in melanocytes, leading to hair graying [27]. Repetitive subcutaneous administrations of SHED-CM showed the protective effects against hair graying, possibly owing to its antioxidative activity mediated by mainly HGF and probably VEGF. This is the first report on the protective effect of SHED-CM against hair graying via its antioxidant activity.

## 2. Materials and Methods

### 2.1. Cell Culture

Immortalized SHED, which was previously established by inducing genes of human telomerase reverse transcriptase, human papillomavirus type 16 E6 and E7, and human B cell-specific Moloney murine leukemia virus integration site 1 [25], was cultured in Dulbecco’s Modified Eagle Medium (DMEM) supplemented with 10% fetal bovine serum (FBS), 100 U/mL penicillin, and 100 μg/mL streptomycin (FUJIFILM Wako Pure Chemical Corporation, Osaka, Japan) at 37 °C in an atmosphere of 5% CO_2_/95% air. Mouse melanoma cell line B16F10 cells (provided by Dr. K. Hirokawa) and mouse keratinocyte cell line PAM212 cells (provided by Dr. S. Tajima) were cultured in DMEM supplemented with 100 U/mL penicillin and 100 μg/mL streptomycin at 37 °C in an atmosphere of 5% CO_2_/95% air.

### 2.2. Mice

C57BL/6 male mice, 6 weeks old, were purchased from Sankyo Labo Service Corporation (Tokyo, Japan). All mice were maintained under pathogen-free conditions, and all animal experiments were approved by the President and the Institutional Animal Care and Use Committee of Tokyo Medical University (Approval numbers: R5-106 and R6-019), and were performed in accordance with institutional, scientific community, and national guidelines for animal experimentation and the Animal Research: Reporting of In Vivo Experiments guidelines.

### 2.3. Preparation of SHED-CM

Approximately 1 × 10^5^ SHED cells were seeded in 10 mL DMEM containing 10% FBS and cultured for 1 week to 80~90% confluence. After washing twice with phosphate-buffered saline and once with serum-free DMEM (Gibco, Grand Island, NY, USA), the cells were further cultured in serum-free DMEM for 72 h. The culture supernatants were then collected, centrifuged at 1750× *g* for 10 min to remove the cell debris, and filtered through a 0.22 µm filter. The culture supernatants were used as the CM and stored at −80 °C until use. The levels of cytokines and growth factors in SHED-CM were reported in our previous paper [25], where the levels of 80 different molecules were analyzed using the multiplex ELISA array. The protein concentration of SHED-CM was determined to be approximately 2.5 mg/mL by the BCA Protein Assay Kit using bovine serum albumin as a standard protein (Takara Bio, Shiga, Japan).

### 2.4. Hair Graying Mouse Model Construction

**Figure 1:**
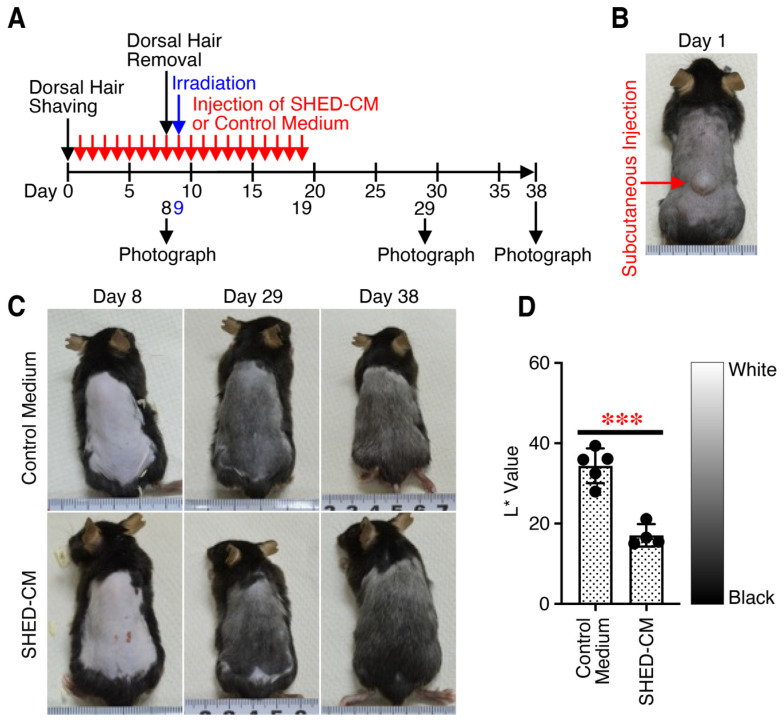
Multiple administrations of SHED-CM suppress hair graying caused by X-ray irradiation. (**A**) Experimental time schedule for X-ray irradiation-induced hair graying and administration of control medium or SHED-CM. (**B**) Representative photograph of the slightly bulging dorsal skin just after subcutaneous administration of SHED-CM. (**C**) Representative photographs of irradiated dorsal skin with time after multiple administrations of control medium or SHED-CM. (**D**) Quantification of hair darkness with the L*a*b* color system. Data are shown as the mean ± SD (*n* = 4–5) and are representative of three independent experiments. *p*-values were determined using unpaired two-tailed Student’s *t*-test. *** *p* < 0.001. In the picture of the measuring tape, the distance between the thick lines is 1 cm.

The hair graying mouse model was developed through X-ray irradiation of C57BL/6 mice after removal of dorsal hair, as reported previously (Figure 1A) [26]. Briefly, 6-week-old C57BL/6 mice were habituated for 1 week, and their dorsal hair was shaved at 7 weeks of age (day 0). Then, 100 µL of SHED-CM or control medium was subcutaneously injected into the dorsal skin from day 1 every day for 1 week. On day 8, the dorsal hair was removed by waxing, in order to induce the anagen hair cycle and, on day 9, the mice were irradiated with a 5 Gy X-ray dose. During this period, 100 µL of SHED-CM or control medium was subcutaneously injected into the dorsal skin every day from day 1 to day 19, and photographs of the dorsal area were taken with time. Hair blackness was assessed approximately 1 month after irradiation and quantified from each photograph with the CIELAB (CIE L*a*b*) color system [28] using Fiji (an expanded version of ImageJ, version 1.53c; National Institutes of Health). The L* value indicates hair brightness, from 0 (black) to 100 (white). The average L* value is shown as the result of the part of dorsal area where the administration was performed.

### 2.5. Immunohistochemical Analysis

Epidermal tissues were removed from mouse dorsal skin, fixed in formalin, and embedded in paraffin. De-paraffinized sections (4 µm) were treated with an autoclave for 20 min at 105 °C in pH 6 antigen retrieval buffer (PerkinElmer, Waltham, MA, USA). Using the N-Histofine MOUSESTAIN KIT (Nichirei Bioscience, Tokyo, Japan), the sections were blocked and incubated with mouse anti-8-hydroxy-2′-deoxyguanosine (8-OHdG) (clone N45.1; Japan Institute for the Control of Aging), followed by incubation with appropriate HRP-labeled polymer-conjugated secondary antibody, according to the manufacturer’s protocol. Immunoreactivity was visualized with 3,3′-diaminobenzidine tetrahydrochloride (Agilent Technologies, Santa Clara, CA, USA). The sections were counterstained with hematoxylin and eosin. The positive areas were quantified using FIJI (Version 1.53c, NIH, Bethesda, MD, USA), and data are reported as relative values.

### 2.6. Measurement of ROS

The ROS levels generated by X-ray irradiation in B16F10 and PAM212 cells were analyzed using the DCFDA Cellular ROS Detection Assay Kit (Abcam, Tokyo, Japan), according to the manufacturer’s protocol. DCFDA is converted by ROS into the highly fluorescent 2′,7′-dichlorofluorescein (DCF). After cells were seeded in complete DMEM supplemented with 10% FBS in an 8-well chamber plate (Fukae Kasei, Hyogo, Japan) and incubated overnight, the cells were pre-incubated with 30% SHED-CM (5% FBS) or control medium (5% FBS) for 3 h. Thereafter, the cells were incubated with 20 μM DCFDA solution for 30 min at 37 °C in dark. After changing the DCFDA solution to 30% SHED-CM (5% FBS) or DMEM medium (5% FBS), X-ray irradiation (30 Gy) was performed. After 1 h, the unfixed cells were stained with Hoechst 33258 (Dojindo, Kumamoto, Japan), and the fluorescence was measured using a confocal laser scanning microscope FluoView FV10 (Olympus, Tokyo, Japan). DCF and Hoechst 33258 signals were detected at Ex/Em = 480/527 nm and Ex/Em = 350/461 nm, respectively. Quantification of the fluorescence intensity was conducted with Fiji (Version 1.53c, NIH).

### 2.7. Cell Viability Assay

B16F10 cells (4 × 10^4^) were seeded onto a 24-well plate in complete DMEM supplemented with 10% FBS and incubated overnight. The cells were then pre-incubated with 30% SHED-CM (5% FBS) or control medium (5% FBS), N-acetyl-L-cysteine (NAC) for 3 h, irradiated by X-ray (30 Gy), and incubated for a further 1 or 24 h. Then, the cells were stained using FITC Annexin V Apoptosis Detection Kit with 7-amino-actinomycin D (7-AAD, BioLegend, San Diego, CA, USA), according to the manufacturer’s protocol. Annexin V^+^ population and 7-AAD^+^ population were detected with a FACSCanto II flow cytometer (BD Biosciences, San Diego, CA, USA) and analyzed using the FlowJo software (version 10: FlowJo).

### 2.8. Immunodepletion

To immunodeplete HGF or VEGF from SHED-CM, immunoprecipitation was performed. Briefly, SHED-CM was incubated with antibody against HGF (clone 24612; R&D Systems, Minneapolis, MN, USA), VEGF (clone R012; Sino Biological, Wayne, PA, USA), or control IgG conjugated to protein G-Sepharose (GE Healthcare, Tokyo, Japan) overnight at 4 °C. After centrifugation, the supernatant was used as SHED-CM depleted of respective cytokines. HGF or VEGF was significantly depleted by the antibody treatment, as determined by ELISA (R&D, Figure S1A,B).

### 2.9. Statistical Analysis

Data are described as the mean  ±  standard deviation (SD) for each group. Statistical analysis was performed with the GraphPad Prism software (version 7.05; GraphPad Software) using the unpaired two-tailed Student’s *t*-test for comparisons of two groups and one-way analysis of variance (ANOVA) with Tukey’s or Dunnett’s multiple comparison test for comparisons involving three groups. *p* < 0.05 was considered to indicate statistical significance.

## 3. Results

### 3.1. Multiple Administrations of SHED-CM Suppress Hair Graying Caused by X-Ray Irradiation

The effect of SHED-CM on hair graying was investigated using a mouse model of hair graying caused by X-ray irradiation. SHED-CM or control medium was subcutaneously injected into the hair-removed dorsal skin every day from day 1 to day 19, with transient subcutaneous swelling observed each time (Figure 1A,B). On day 9, the mice were irradiated with X-rays after the removal of hair via waxing on day 8. The color of the dorsal hair was observed, and it was photographed at various time points. After X-ray irradiation, the hair color gradually turned black. The hair of mice injected with SHED-CM appeared to be blacker than that of mice administered control medium on days 29 and 38 (Figure 1C). The L* value, which indicates hair brightness, was quantified with the L*a*b* color system on day 38. The hair color of mice injected with SHED-CM was significantly blacker than that of mice administered control medium (Figure 1D). These results suggest that multiple administrations of SHED-CM suppress hair graying caused by X-ray irradiation.

### 3.2. Multiple Administrations of SHED-CM Reduce ROS Generation Caused by X-Ray Irradiation in the Skin

**Figure 2:**
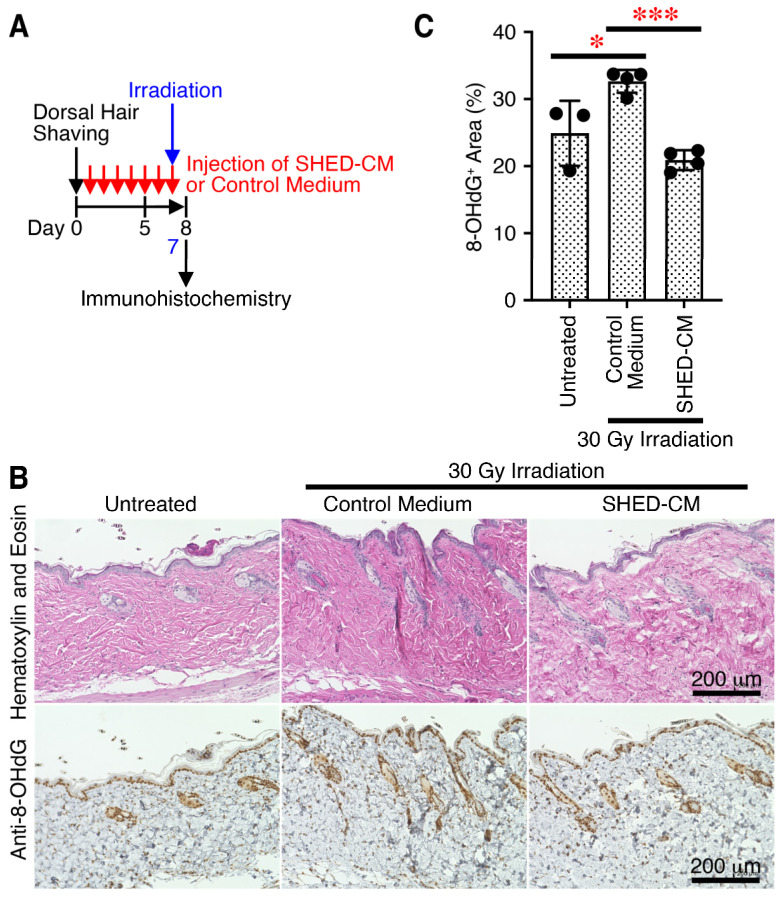
Multiple administrations of SHED-CM reduce ROS generation caused by X-ray irradiation in the skin. (**A**) Experimental time schedule for X-ray irradiation-caused hair graying and administrations of control medium or SHED-CM. (**B**) Dorsal skin tissues were removed on day 8 and immunohistochemically analyzed for expression of the oxidative stress marker 8-OHdG through counterstaining with hematoxylin and eosin. Representative photographs of their expression are shown. Subcutaneous adipose tissue, sebaceous gland, hair bulb, dermis, and epidermis were positively stained. (**C**) Positive areas for 8-OHdG were calculated using FIJI. Data are shown as the mean ± SD (*n* = 3–4) and are representative of three independent experiments. *p*-values were determined by one-way analysis of variance with the Tukey’s multiple comparisons test. * *p* < 0.05, *** *p* < 0.001.

X-ray irradiation generates oxidative stress, ROS generation, and resultant DNA damage, eventually leading to hair graying [4,26,29]. ROS cause oxidative damage to nucleic acids, proteins, and lipids, and in vivo, 8-OHdG is the major product of DNA damage caused by ROS [30]. To explore the molecular mechanisms by which SHED-CM suppresses hair graying, immunohistochemical analysis using anti-8-OHdG was carried out using tissues of the dorsal skin obtained on day 8 (Figure 2A). X-ray irradiation greatly augmented the amount of 8-OHdG^+^ damaged DNA, and administration of SHED-CM significantly reduced it, compared to the effect of control medium (Figure 2B,C). These results suggest that multiple administrations of SHED-CM reduce the ROS generation caused by X-ray irradiation in the dorsal skin.

### 3.3. SHED-CM Protects Mouse Melanoma Cell Line B16F10 Cells from Cell Death Caused by X-Ray Irradiation

**Figure 3:**
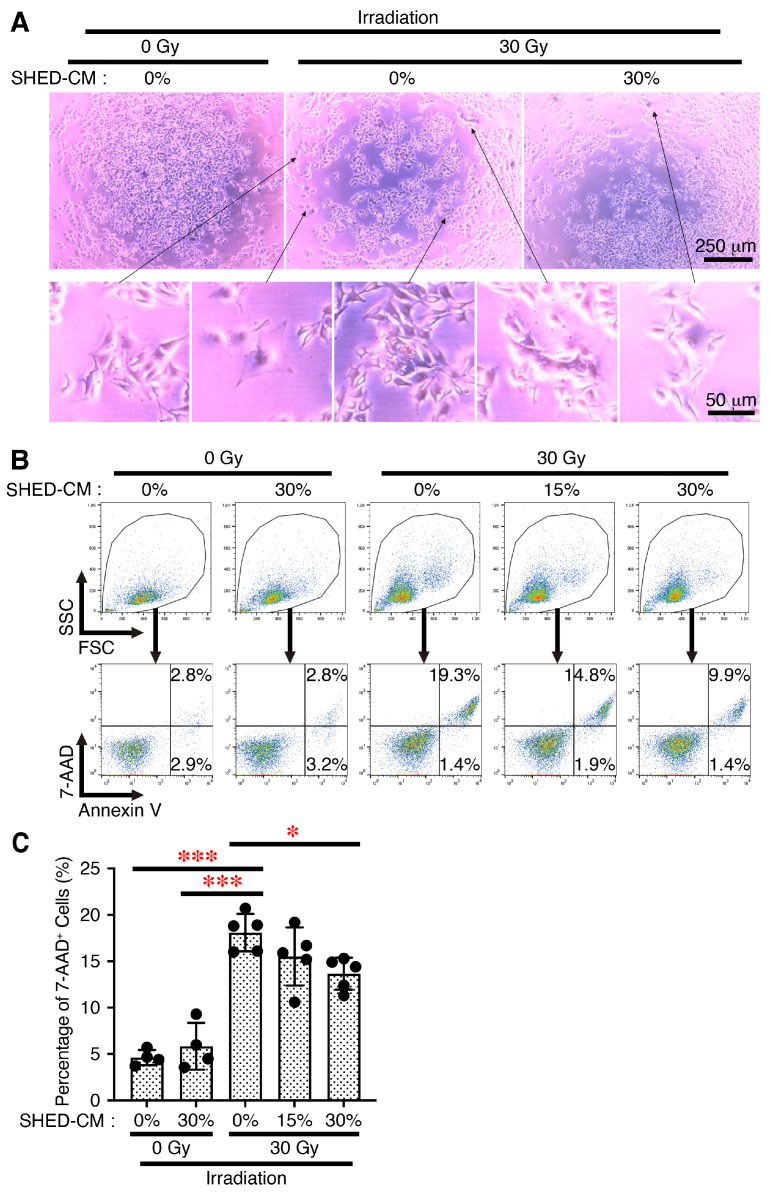
SHED-CM protects mouse melanoma B16F10 cells from cell death caused by X-ray irradiation. (**A**) Mouse melanoma B16F10 cells were pre-incubated with SHED-CM (0, 15, and 30%) for 3 h, and then irradiated by 30 Gy X-ray. After 1 h, the cells were photographed under a microscope, and representative photographs are shown with several arrows pointing at necrotic cells. Cells were then stained with annexin V (FITC) and 7-AAD (PerCP-Cy5.5) and analyzed with FACS. (**B**) Representative dot-plots are shown. (**C**) The percentage of necrotic cells positive for 7-AAD was calculated. Data are shown as the mean ± SD (*n* = 4–5), and are representative of three independent experiments. *p*-values were determined by one-way analysis of variance with Dunnett’s multiple comparison test. * *p* < 0.05, *** *p* < 0.001.

We next explored the effects of SHED-CM on X-ray irradiation-induced cell death, which is highly mediated by the generation of ROS, in mouse melanoma cell line B16F10 cells. After the pre-incubation of B16F10 cells with control medium or SHED-CM, their susceptibility to X-ray irradiation-induced cell death was determined through staining with annexin V and 7-AAD. A visual analysis of the apparent appearance under a microscope revealed that X-ray irradiation greatly increased the number of necrotic-like dead cells in control medium (Figure 3A). In contrast, SHED-CM greatly reduced the number of such necrotic-like dead cells. Moreover, FACS analysis revealed that X-ray irradiation greatly increased the percentage of necrotic cells positive for 7-AAD in B16F10 cells pre-incubated with control medium (Figure 3B,C). In contrast, the pre-incubation of B16F10 cells with SHED-CM significantly reduced the percentage of 7-AAD^+^ necrotic cells in a dose-dependent manner. These results suggest that SHED-CM protects B16F10 cells from cell death caused by X-ray irradiation.

### 3.4. SHED-CM Inhibits ROS Generation Caused by X-Ray Irradiation in an HGF- and Probably VEGF-Dependent Manner

**Figure 4:**
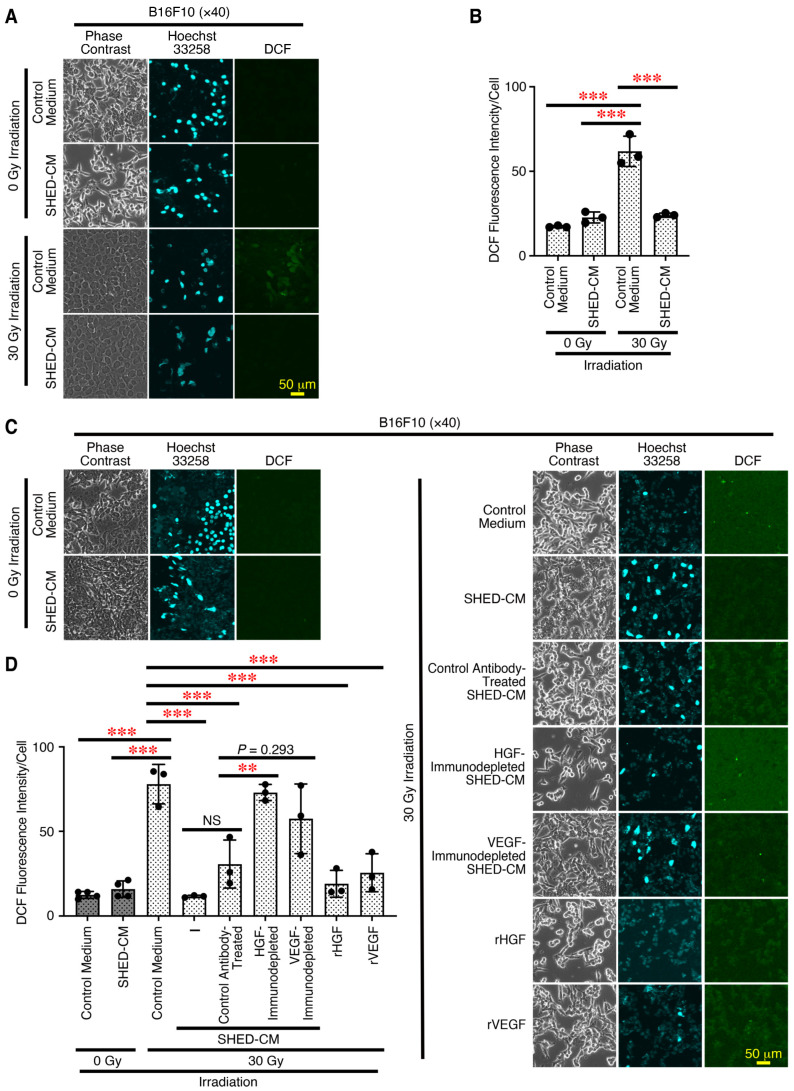
SHED-CM inhibits ROS generation caused by X-ray irradiation in an HGF- and probably VEGF-dependent manner. Mouse melanoma B16F10 cells were pre-incubated with control medium or 30% SHED-CM (**A**,**B**), control antibody-treated SHED-CM, HGF- or VEGF-immunodepleted SHED-CM, rHGF (10 ng/mL), rVEGF (10 ng/mL) (**C**,**D**) for 3 h. These cells were then treated with DCFDA, irradiated by 30 Gy X-ray, and counterstained with Hoechst 33258. Representative photographs of the ROS generation are shown (**A**,**C**). The fluorescence intensity of DCF and Hoechst 33258 was quantified using FIJI and calculated as arbitrary units per cell (**B**,**D**). Data are shown as the mean ± SD (*n* = 3), and are representative of two independent experiments. *p*-values were determined by one-way analysis of variance with Tukey’s multiple comparisons test. ** *p* < 0.01, *** *p* < 0.001. NS, not significant.

Hair follicular keratinocyte stem cells maintain a niche for McSCs in the hair bulge [6]. X-ray irradiation induces aberrant proliferation and differentiation of McSCs, but does not directly affect the McSCs, and causes deficiency in the niche function of hair follicular keratinocyte stem cells [4,5]. Therefore, the effects of SHED-CM on X-ray irradiation-caused ROS generation in both melanocytes and keratinocytes (using the mouse cell lines B16F10 and PAM212, respectively) were examined next. Without any X-ray irradiation, basal ROS generation was observed in B16F10 cells. However, 30 Gy X-ray irradiation of B16F10 cells pre-incubated with control medium greatly increased ROS generation. In contrast, X-ray irradiation of B16F10 cells pre-incubated with SHED-CM greatly suppressed the ROS generation to the basal level without any X-ray irradiation (Figure 4A,B). Similarly, 30 Gy X-ray irradiation of PAM212 cells pre-incubated with control medium greatly increased ROS generation, whereas that with SHED-CM greatly suppressed ROS generation to the basal level (Figure S2A,B). These results suggest that SHED-CM inhibits the ROS generation caused by X-ray irradiation in both B16F10 melanoma cells and PAM212 mouse keratinocyte cells.

We previously demonstrated that SHED-CM has potent therapeutic effects in a mouse model of the formation of pressure ulcer induced by cutaneous ischemia–reperfusion injury, which highly depend on antioxidative and angiogenic activities, mainly via HGF and VEGF [25]. Therefore, we next explored the role of these cytokines in the suppressive effects of SHED-CM on the ROS generation caused by X-ray irradiation. HGF or VEGF in the SHED-CM were immunodepleted using their respective specific antibodies, whose depletion was confirmed by ELISA (Figure S1A,B). SHED-CM treated with control antibody significantly inhibited the ROS generation caused by X-ray irradiation (Figure 4C,D). In contrast, SHED-CM immunodepleted of HGF greatly reversed the inhibitory effect, while SHED-CM immunodepleted of VEGF tended to reverse the inhibitory effect, but this tendency was not significant (Figure 4C,D). Moreover, the addition of rHGF or rVEGF greatly inhibited ROS generation. These results suggest that SHED-CM inhibits the ROS generation caused by X-ray irradiation in an HGF- and probably VEGF-dependent manner.

### 3.5. SHED-CM Protects Mouse Melanoma Cell Line B16F10 Cells from Cell Death Caused by X-Ray Irradiation in an HGF- and VEGF-Dependent Manner

**Figure 5:**
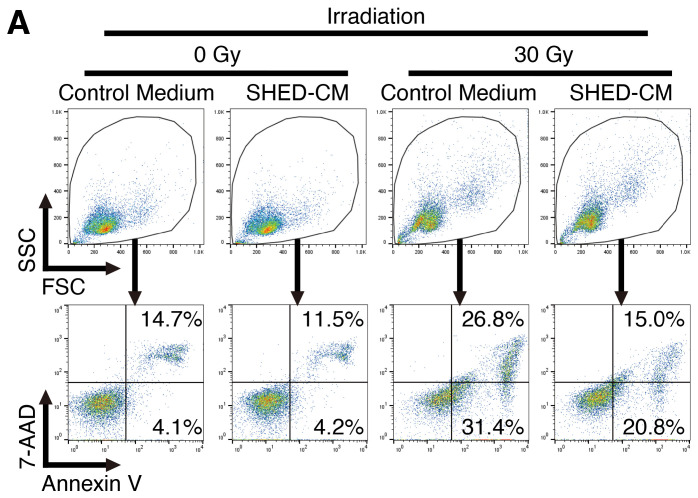

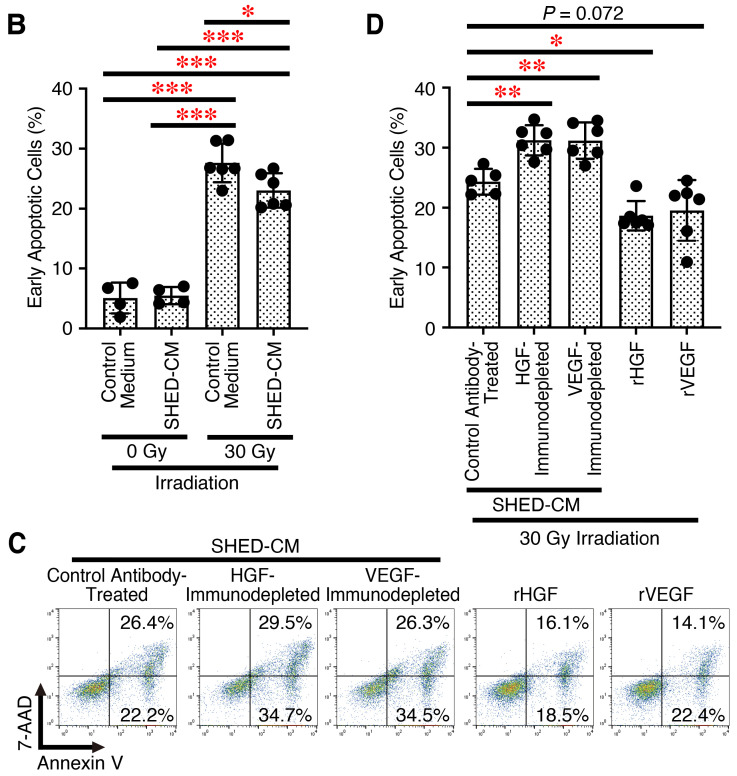
SHED-CM protects mouse melanoma B16F10 cells from cell death caused by X-ray irradiation in an HGF- and VEGF-dependent manner. (**A**) Mouse melanoma B16F10 cells were pre-incubated with control medium or 30% SHED-CM (**A**,**B**), control antibody-treated SHED-CM, HGF- or VEGF-immunodepleted SHED-CM, rHGF (10 ng/mL), and rVEGF (10 ng/mL) (**C**,**D**) for 3 h, and then irradiated by 30 Gy X-ray. After 24 h, the cells were stained with annexin V and 7-AAD, and analyzed with FACS. Representative dot-plots are shown (**A**,**C**). The percentage of early apoptotic cells positive for annexin V but negative for 7-AAD was calculated (**B**,**D**). Data are shown as the mean ± SD (*n* = 4–6), and are representative of three independent experiments. *p*-values were determined by one-way analysis of variance with Tukey’s (**B**) and Dunnett’s (**D**) multiple comparison test. * *p* < 0.05, ** *p* < 0.01, *** *p* < 0.001.

Finally, we explored the role of HGF and VEGF in the protective effects of SHED-CM against cell death caused by X-ray irradiation in B16F10 cells. After pre-incubation of B16F10 cells with control medium or SHED-CM, control antibody-treated SHED-CM, HGF- or VEGF-immunodepleted SHED-CM, rHGF, and rVEGF, their susceptibilities to 30 Gy X-ray irradiation-induced cell death were determined 24 h after irradiation by staining with annexin V and 7-AAD, followed by FACS analysis. X-ray irradiation greatly increased the percentage of early apoptotic cells positive for 7-AAD but negative for annexin V in B16F10 cells pre-incubated with control medium (Figure 5A,B). In contrast, the pre-incubation of B16F10 cells with SHED-CM slightly but significantly reduced the percentage of early apoptotic cells (Figure 5A,B). Moreover, the depletion of HGF or VEGF from SHED-CM significantly increased the percentage of early apoptotic cells (Figure 5C,D). Consistent with these results, rHGF or rVEGF significantly or almost significantly reduced the percentage of early apoptotic cells, respectively (Figure 5C,D). These results suggest that SHED-CM protects B16F10 cells from cell death caused by X-ray irradiation in an HGF- and VEGF-dependent manner.

## 4. Discussion

In response to stimuli such as ultraviolet irradiation and other stresses, keratinocytes and fibroblasts secrete several paracrine factors, including α-melanocyte-stimulating hormone, adrenocorticotropic hormone, stem cell factor, endothelin-1, and Wingless-related integration site (WNT), which activate multiple downstream signaling pathways through their corresponding receptors on melanocytes, leading to the activation of microphthalmia-associated transcription factor—the critical transcriptional factor for melanogenesis [31,32]. Transgenic mice expressing human cytokeratin 14 promoter driving cytokine cDNAs of stem cell factor, HGF, and endothelin-3 were previously shown to prevent hair graying induced by repeated plucking or shaving of trunk hairs [33]. Moreover, inflammatory cytokines such as interleukin (IL)-18, IL-33, granulocyte-macrophage colony-stimulating factor, and IL-1α have been demonstrated to promote melanogenesis [34]. As SHED-CM contains several cytokines, including HGF and stem cell factor [25], the preventive effects of SHED-CM against hair graying could be highly attributed to the synergistic effects of these cytokines.

Tissue regeneration highly depends on the ability of adult stem cells such as MSCs to differentiate, which is generally controlled in a unidirectional manner according to the hierarchical model originally established for hematopoietic stem cells [35]. This model suggests two asymmetric fates: one is the self-renewal ability to maintain stem cells, while the other is multipotency, which ultimately gives rise to functionally differentiated cells through transit-amplifying progeny [36]. However, McSCs become exhausted earlier than hair follicle stem cells, resulting in hair graying with age. Therefore, different from the unidirectional hierarchical model, a new reversible differentiation model has been proposed, in which the de-differentiation of McSCs plays an important role in the homeostatic maintenance of stem cells [37]. The hair cycle consists of three stages: anagen, catagen, and telogen, corresponding to regeneration, degeneration, and rest stage of hair follicles, respectively. During the telogen stage, McSCs in the hair germ are in a stem-like state, and at the onset of anagen, McSCs undergo activation and differentiate into a transit-amplifying intermediate state through WNT activation. Subsequently, the cells either fully differentiate into terminal melanocytes in the hair bulbs or de-differentiate into a stem-like state upon translocation into the WNT-negative bulge, consequently homing back to the hair germ [37]. Although SHED-CM contains several cytokines bearing the potential to act on these stem cells, including HGF, basic fibroblast growth factor, keratinocyte growth factor, etc. [25], whether SHED-CM has the ability to act on McSCs, protect them from exhaustion, and maintain their stemness via these cytokines remains to be elucidated.

Evidence showing a direct relationship between hair graying and psychological stress has recently been reported [38]. Upon psychological stress, activation of the sympathetic nerves greatly induces release of the neurotransmitter noradrenaline (norepinephrine), which then innervates the quiescent McSCs to proliferate and differentiate, resulting in their depletion in a manner independent of the immune response or adrenal stress hormones. Schwann cells are glial cells that produce a multilayered myelin sheath to insulate nerves for efficient impulse conduction at the peripheral nerve axons [39,40]. The regeneration of peripheral nerves highly relies on the plasticity of Schwann cells. Upon nerve injury, mature Schwann cells undergo de-differentiation toward a cell phenotype resembling immature Schwann cells [39,41,42]. SHED was derived from the dental pulp of deciduous teeth and can differentiate into multiple cells with characteristics similar to mesoderm, endoderm, and ectoderm cells, including neural-like cells, even in vitro [43]. Notably, all melanocytes, Schwann cells, and dental pulp stem cells developmentally arise from the neural crest and have similar characteristics—including high plasticity—in adults. In particular, melanocytes and Schwann cells share the same progenitor: Schwann cell precursor cells [44]. Due to their high plasticity, upon peripheral nerve injury, Schwann cells have been reported to de-differentiate and transdifferentiate into melanocytes, resulting in pigmentation of the skin dermis [45]. Thus, these three cells are very closely related and, therefore, SHED-CM may be very effective on not only melanocytes but also Schwann cells, the possibility of which is currently under investigation.

We recently demonstrated that SHED-CM has antioxidative and angiogenic activities in a mouse model of the formation of pressure ulcer induced by cutaneous ischemia–reperfusion injury [25]. Consistent with this report, the present results revealed that SHED-CM has protective effects against hair graying in a mouse model caused by X-ray irradiation, presumably due to its antioxidative activity. The ability of MSCs to attenuate tissue injury through the suppression of ROS generation has been demonstrated in several disease models [46,47]. The antioxidative effects are mediated in a paracrine manner via secreted factors, such as cytokines, growth factors, and exosomes, as well as in a cell-contact-dependent manner. The possible mechanisms include scavenging of free radicals, enhancement of endogenous antioxidant defenses, modulation of the inflammatory responses, and augmentation of cellular respiration and mitochondrial functions. Exosomes have also been reported to directly reduce excessive ROS, promote intracellular antioxidant stress defense, modulate the immune system by inhibiting excessive ROS, and alter mitochondrial performance [47]. Whether exosomes in SHED-CM are also critical for the protective effects of SHED-CM against hair graying remains to be investigated. Recently, several problems have emerged with many of the commonly used approaches for measuring ROS and oxidative damage [48]. For instance, DCFDA is a widely used fluorescent probe; it has recently been pointed out that it is not specific for any particular ROS and that there are several issues, including selectivity, quantification, linearity of response, and susceptibility to artefacts. Oxidative modifications of DNA are often used as biomarkers of oxidative damage, and immunohistochemical analysis using an antibody to 8-OHdG has been widely used to identify cells whose DNA is oxidized in vivo. In the present study, we also showed that the X-ray-induced cell death in B16F10 cells was inhibited by the antioxidant NAC in a dose-dependent manner (Figure S3), supporting that SHED-CM indeed has antioxidant activity. Additional investigations into factors such as telomere damage and oxidized mitochondrial DNA would further strengthen our conclusion.

As there are currently no effective treatments against hair graying [9], the cell-free therapy using SHED-CM may be a promising treatment for this purpose. Although we used multiple doses and continuous administrations of SHED-CM in this study, it would generally be very difficult to extrapolate the mouse results directly to humans. In fact, we have not optimized the protocol, including the number of administrations, dose, frequency, and duration of SHED-CM, together with its cost-effectiveness. It may be necessary to use a device to deliver the SHED-CM continuously to the skin. In addition, one of the primary concerns with immortalized MSCs is tumorigenicity, especially since immortalization processes of overexpression of human telomerase reverse transcriptase and oncogenes might increase the risk of malignant transformation [49]. By using only the bioactive factors secreted by immortalized MSCs, however, the risk of tumor formation or other complications is significantly reduced compared to administering immortalized cells directly. In addition, even transformed cells from immortalized MSCs do not directly release cancer cells into the conditioned media. In fact, we have never observed any tumor formation in mice following multiple intravenous, intraperitoneal, intradermal, subcutaneous and intranasal administrations of SHED-CM. However, the quality and composition of the CM may change due to genetic instability during prolonged culture depending on the culture conditions. This may affect the therapeutic properties and safety of the derived products. Therefore, rigorous safety testing, such as with the soft agar colony forming assay [50], and monitoring for chromosomal abnormalities by fluorescence in situ hybridization (FISH) [51] or karyotyping [52,53], is needed to ensure safety. Quality control, including ensuring the purity, stability, potency, and safety of the products, will be essential, especially as immortalized MSCs are cultured for long periods of time. Although many issues would thus need to be addressed before it could be introduced into practical medicine, immortalized SHED could serve as an ideal cell source for production of such CM, as it grows vigorously and indefinitely, consistently and stably secreting various cytokines [25,49]. Thus, the CM of immortalized SHED may pave an avenue for the creation of a new type of cell-free regenerative medicine against hair graying, although further studies on its effectiveness and safety are warranted.

## Data Availability

Data is contained within the article.

## Supplementary Materials

The following supporting information can be downloaded at: https://www.mdpi.com/article/10.3390/antiox14010109/s1, Figure S1: Immunodepletion of HGF or VEGF from SHED-CM; Figure S2: SHED-CM inhibits ROS generation caused by X-ray irradiation in mouse keratinocyte cell line HaCaT cells; Figure S3: X-ray-induced cell death is mediated by ROS generation in B16F10 cells.

## Abbreviations

The following abbreviations are used in this manuscript:

7-AAD	7-amino-actinomycin D
ANOVA	analysis of variance
CM	conditioned medium
DCF	2′,7′-dichlorofluorescein
DMEM	Dulbecco’s Modified Eagle Medium
FBS	fetal bovine serum
HGF	hepatocyte growth factor
IL	interleukin
McSCs	melanocyte stem cells
MSCs	mesenchymal stem cells
NAC	N-acetyl-L-cysteine
8-OHdG	8-hydroxy-2′-deoxyguanosine
ROS	reactive oxygen species
SD	standard deviation
SHED	stem cell line from human exfoliated deciduous teeth
VEGF	vascular endothelial growth factor
WNT	Wingless-related integration site
