# Supplementary material for: Protective Effect of Conditioned Medium of Immortalized Human Stem Cells from Exfoliated Deciduous Teeth Against Hair Graying Caused by X-Ray Irradiation via Its Antioxidative Activity
Source: Antioxidants (Basel). 2025 Jan 18;14(1):109. doi: 10.3390/antiox14010109 (PMC11760446; doi:10.3390/antiox14010109)
Supplement: Supplementary file 1 [file antioxidants-14-00109-s001.zip › antioxidants-3418861-supplementary.pdf]

Supplementary Material

Article in Antioxidants

Protective Effect of Conditioned Medium of Immortalized Human Stem Cells from Exfoliated Deciduous Teeth against Hair Graying Caused by X-Ray Irradiation via Its Antioxidative Activity

Yasuhiro Katahira [1], Eri Horio [1], Natsuki Yamaguchi [1], Jukito Sonoda [1], Miu Yamagishi [1], Satomi Miyakawa [1], Fumihiro Murakami [1], Hideaki Hasegawa[1], Izuru Mizoguchi [1], and Takayuki Yoshimoto [1,*]

[1]Department of Immunoregulation, Institute of Medical Science, Tokyo Medical University, 6-1-1 Shinjuku, Shinjuku-ku, Tokyo 160-8402, Japan

* Correspondence: yoshimot@tokyo-med.ac.jp; Tel.: +81-3-3351-6141 (ext. 431)

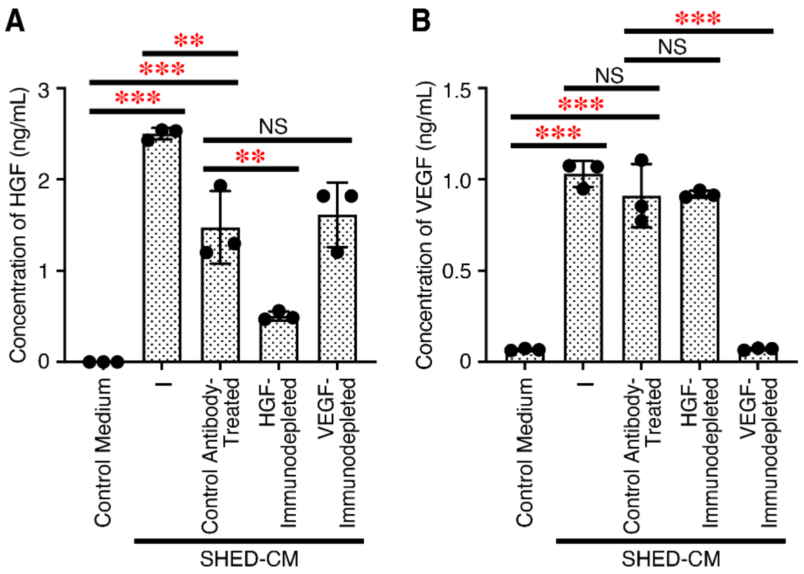

Supplementary Figure S1. Immunodepletion of HGF or VEGF from SHED-CM. SHED-CM was immunodepleted by incubating with antibody against HGF, VEGF, or control IgG conjugated to protein G-Sepharose overnight at 4°C. After centrifugation, the supernatant was used as SHED-CM depleted of respective cytokines, whose concentrations of HGF (A) and VEGF (B) were determined by ELISA. Data are shown as the mean ± SD ($n = 3$), and are representative of two independent experiments. P-values were determined by one-way analysis of variance with Tukey's multiple comparisons test. ** $P < 0.01$, *** $P < 0.001$. NS, not significant.

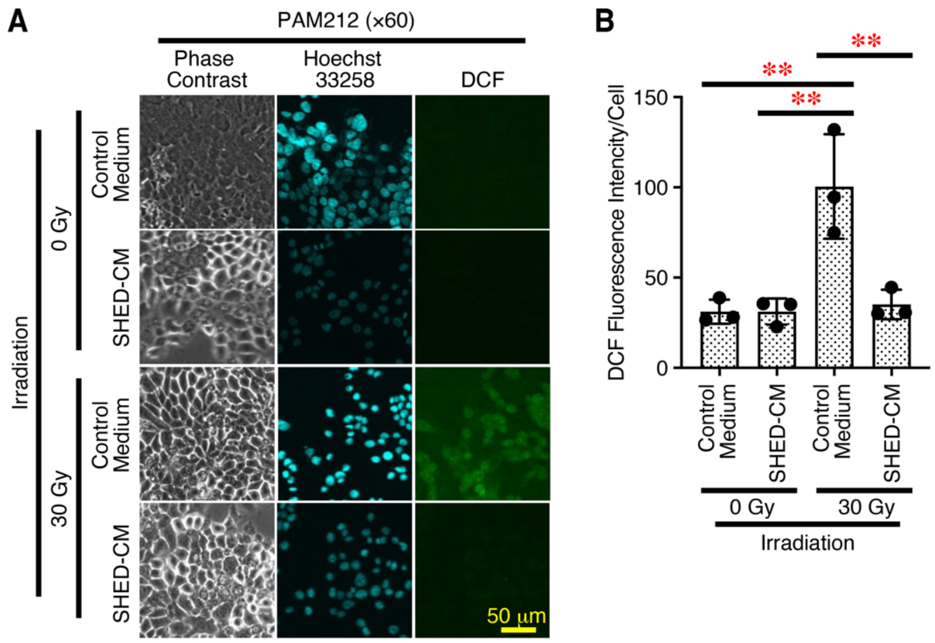

Supplementary Figure S2. SHED-CM inhibits ROS generation caused by X-ray irradiation in mouse keratinocyte cell line PAM212 cells. PAM212 cells were pre-incubated with control medium or 30% SHED-CM (A,B) for 3 h. These cells were then treated with DCFDA, irradiated by 30 Gy X-ray, and counterstained with Hoechst 33258. Representative photographs of the ROS generation are shown (A). The fluorescence intensity of DCF and Hoechst 33258 was quantified using FIJI and calculated as arbitrary unit per cell (B). Data are shown as the mean ± SD (n = 3), and are representative of two independent experiments. P-values were determined by one-way analysis of variance with Dunnett's multiple comparisons test. ** $P < 0.01$.

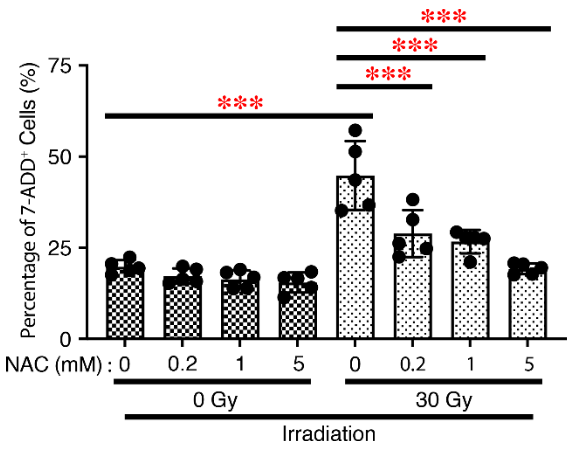

Supplementary Figure S3. X-ray-induced cell death is mediated by ROS generation in B16F10 cells. Mouse melanoma B16F10 cells were pre-incubated with the antioxidant NAC (0, 0.2, 1.0, and 5.0 mM) for 3 h, and then irradiated by 30 Gy X-ray. After 1 h, cells were then stained with annexin V and 7-AAD, and analyzed with FACS. The percentage of necrotic cells positive for 7-AAD was calculated. Data are shown as the mean ± SD ($n = 5$), and are representative of four independent experiments. *P*-values were determined by one-way analysis of variance with Tukey's multiple comparison test. *** $P < 0.001$.
